# Local association of *Trypanosoma cruzi* chronic infection foci and enteric neuropathic lesions at the tissue micro-domain scale

**DOI:** 10.1371/journal.ppat.1009864

**Published:** 2021-08-23

**Authors:** Archie A. Khan, Harry C. Langston, Fernanda C. Costa, Francisco Olmo, Martin C. Taylor, Conor J. McCann, John M. Kelly, Michael D. Lewis

**Affiliations:** 1 Department of Infection Biology, London School of Hygiene and Tropical Medicine, London, United Kingdom; 2 Stem Cells and Regenerative Medicine, University College London, Institute of Child Health, London, United Kingdom; UNITED KINGDOM

## Abstract

Digestive Chagas disease (DCD) is an enteric neuropathy caused by *Trypanosoma cruzi* infection. The mechanism of pathogenesis is poorly understood and the lack of a robust, predictive animal model has held back research. We screened a series of mouse models using gastrointestinal tracer assays and *in vivo* infection imaging systems to discover a subset exhibiting chronic digestive transit dysfunction and significant retention of faeces in both sated and fasted conditions. The colon was a specific site of both tissue parasite persistence, delayed transit and dramatic loss of myenteric neurons as revealed by whole-mount immunofluorescence analysis. DCD mice therefore recapitulated key clinical manifestations of human disease. We also exploited dual reporter transgenic parasites to home in on locations of rare chronic infection foci in the colon by *ex vivo* bioluminescence imaging and then used fluorescence imaging in tissue microdomains to reveal co-localisation of infection and enteric nervous system lesions. This indicates that long-term *T*. *cruzi*-host interactions in the colon drive DCD pathogenesis, suggesting that the efficacy of anti-parasitic chemotherapy against chronic disease progression warrants further pre-clinical investigation.

## Author summary

Chagas disease (American trypanosomiasis) is caused by the protozoan parasite *Trypanosoma cruzi*. Chagas disease has two types, the cardiac form and the digestive form; some patients have symptoms of both. How the parasite causes digestive disease is poorly understood. It is known that damage to the gut’s nervous system is an important factor, but it has been unclear exactly where and when this damage occurs during the course of an infection and also why only a subset of infected people suffer from this outcome. We studied infections in mice and found certain combinations of strains of parasites and mice that exhibited symptoms similar to human digestive Chagas patients, including a problem with peristalsis that localised specifically to the colon. Using parasites that were genetically engineered to emit both bioluminescent and fluorescent light, we tracked infections over time and were able to analyse rare infected cells deep within the muscle tissue of the wall of the colon. We found evidence of damaged neurons in the same location as these infection foci over 6 months after initial infection. Our results show that digestive Chagas disease probably develops as a result of chronic infection and inflammation, which potentially changes approaches to treatment.

## Introduction

Chagas disease (CD) is caused by infection with the protozoan parasite *Trypanosoma cruzi*, which affects approximately 6 million people. There are two principal forms of CD, cardiac and digestive. The most prevalent cardiac presentations include myocarditis, fibrosis, arrhythmias, microvascular abnormalities, progressive heart failure and sudden death [1]. Cardiac CD has been the subject of intensive experimental research and many predictive animal models are available to support translation into the clinic. Human digestive CD (DCD) is characterised by progressive dilatation and dysfunction of sections of the GI tract [2,3]. Symptoms include achalasia, abdominal pain, constipation and faecaloma. Eventually, massive organ dilatation results in megasyndromes, usually of the colon and/or oesophagus. Dilatation is associated with loss of enteric neurons leading to peristaltic paralysis and smooth muscle hypertrophy. Treatments are largely limited to dietary and surgical interventions [4]. The lack of a robust small animal model of enteric CD has been a major block on basic and translational research.

Of symptomatic CD patients, ~65% have cardiomyopathy, 30% enteropathy and 5% have both, with digestive disease most common in Bolivia, Chile, Argentina and Brazil [1]. Anti-parasitic chemotherapy has not been considered justifiable for *T*. *cruzi-*positive individuals with digestive symptoms, but normal heart function, as no clinical trials have addressed treatment efficacy in the context of digestive outcomes [5]. Challenges for clinical trials include the highly variable and unpredictable clinical outcomes of *T*. *cruzi* infection, the extended time frame for disease development and, especially in the context of DCD, a paucity of experimental data [6]. Molecular and cellular explanations of DCD pathogenesis also lag far behind the advances made for Chagas cardiomyopathy.

The lack of progress in developing treatments for DCD may also be connected to the idea that megasyndromes result from irreversible enteric denervation, specifically during the *acute* phase of infection [7,8], in which anti-parasitic inflammatory responses are thought to cause iNOS-dependent collateral damage to neurons, leading to aganglionosis [7,9]. Further age-related nerve degeneration was posited to gradually unmask these parasite-driven losses, leading to progressive organ dysfunction on a timescale of years to decades [7]. However, the frequent detection of *T*. *cruzi* and signs of active inflammation in oesophageal and colonic tissues from patients circumstantially suggests that chronic parasite persistence may contribute to disease development [10–17]. Furthermore, experimental bioluminescence imaging and tissue PCR studies in mice revealed that the GI tract is a major long-term reservoir of *T*. *cruzi* infection [18–22]. Adult enteric neurogenesis has been described in response to chemically-mediated tissue injury [23] and in the steady state [24]. A series of advances has also highlighted previously unappreciated levels of interconnectedness between the gut’s immune and nervous systems [25–27]. We therefore sought to develop murine DCD models suitable to address the hypothesis that host-parasite interactions in the chronically infected gut might impact continuously on the enteric nervous system (ENS) and musculature to drive disease pathogenesis.

Here, we studied a series of parasite and mouse strain combinations to identify several models with significant digestive motility dysfunction. Using a combination of bioluminescence and fluorescence *in vivo* and *ex vivo* imaging techniques, we demonstrate that chronic *T*. *cruzi* persistence, gut motility delay and enteric neuronal damage are co-localised within discrete foci in the colonic muscularis. This indicates that DCD tissue pathology and transit dysfunction are likely driven by *T*. *cruzi* persistence in the colon and the associated chronic inflammatory response. DCD should therefore be considered as potentially preventable by anti-parasitic chemotherapy. It also opens the way to investigate the molecular and cellular basis of pathogenesis and *T*. *cruzi* immune evasion.

## Results

### A subset of *T*. *cruzi* mouse infection models display chronic digestive transit time delays

**Fig 1:**
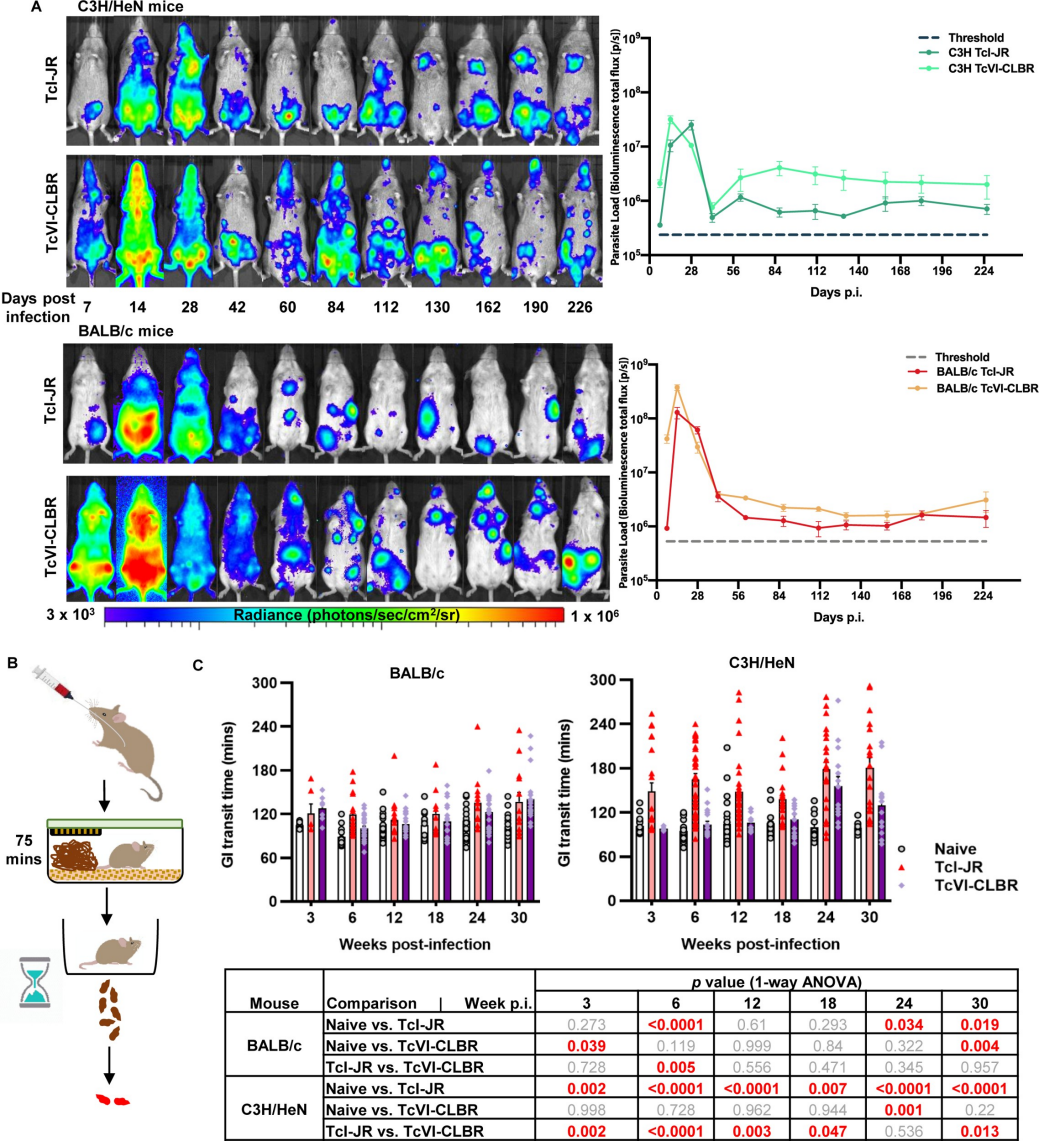
Bioluminescence imaging *T*. *cruzi* infection models and digestive transit dysfunction screen. **A.** Ventral images of female C3H/HeN (top panels) and BALB/c mice (bottom panels) representing TcI-JR (1^st^ and 3^rd^ panel) and TcVI-CLBR (2^nd^ and 4^th^ panel) course of infection. Images were captured using *in vivo* bioluminescence imaging. Overlaid log-scale pseudocolour heat maps are representative of bioluminescence intensity; the log-scale range is indicated in units of radiance. Adjacent line plots show parasite load represented as average bioluminescence of TcI-JR C3H/HeN (*n* = 10–24), TcVI-CLBR C3H/HeN (*n* = 5–12), TcI-JR BALB/c (*n* = 5–12) and TcVI-CLBR BALB/c (*n* = 9–22) infected mice against days post infection (p.i.). Limit of detection of bioluminescence is indicated as threshold by dashed line. **B.** Schematic diagram of the carmine red-dye assay to measure gastrointestinal (GI) transit time delay in mice. **C.** Bar plots show GI transit time vs. weeks post-infection (p.i.) of BALB/c (left) and C3H/HeN (right) mice in the following groups: naive control BALB/c (*n* = 8–18), TcI-JR BALB/c (*n* = 6–17), TcVI-CLBR BALB/c (*n* = 10–29), naive control C3H/HeN (*n* = 12–35), TcI-JR C3H/HeN (*n* = 18–38) and TcVI-CLBR C3H/HeN (*n* = 6–17). Table (bottom) summarises statistical comparisons of GI transit time delay between groups. All statistically significant values are highlighted (red). Data are expressed as mean ± SEM. Statistical significance was tested using one way ANOVA followed by Tukey’s HSD test.

We previously developed a series of mouse models of *T*. *cruzi* infection based on parasites transgenically expressing the luciferase variant *Ppy*RE9h, which serves as an orange-red light emitting *in vivo* reporter protein [28]. Host-parasite combinations of BALB/c and C3H/HeN mice and TcVI-CL Brener (CLBR) and TcI-JR strain parasites permit long-term tracking of the course and distribution of infections in individual animals (Fig 1A). These models, which exhibit a spectrum of Chagas heart disease severities [19], were screened for gastrointestinal (GI) transit time delays, a common feature of DCD, by oral feeding of a red dye tracer (carmine). Three of the four host-parasite combinations took significantly longer than control uninfected mice to pass the tracer at acute phase, 3 weeks post-infection (p.i.), and/or at 6 weeks p.i. transition phase (Fig 1B). During the early chronic phase, 12 and 18 weeks p.i., only the TcI-JR-infected C3H mice displayed the delay phenotype, which became markedly more severe as the infection developed into the later chronic phase at 24 and 30 weeks p.i. Milder, but still significant transit delay phenotypes also emerged in the other three models.

*T*. *cruzi* as a species encompasses a high level of genetic diversity structured across six major lineages [29–31]. To test whether and at what level the strong digestive transit delay phenotype in C3H mice was conserved, we tested a further eight *T*. *cruzi* strains from five lineages (4x TcI, 1x TcII, 1x TcIII, 1x TcIV and 1x TcVI) using the carmine transit assay (S1 Fig). Two more strains were identified showing evidence of delayed transit: TcI-SN3 and TcVI-Peru. This type of pathology is therefore a relatively rare, strain-specific trait in *T*. *cruzi*. It occurs in both TcI and TcVI strains, but is not conserved within lineages.

### Parasite persistence within the GI tract

**Fig 2:**
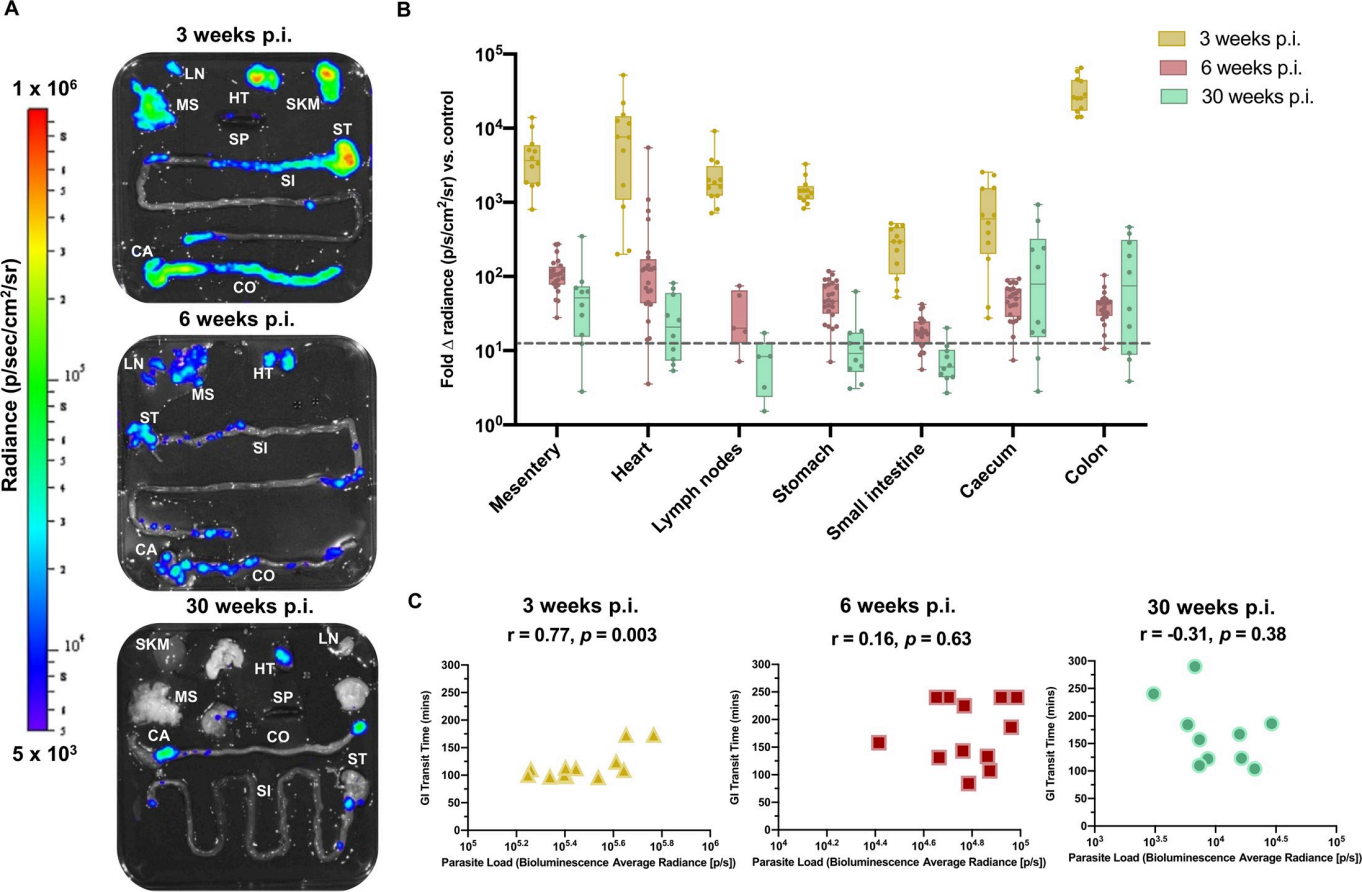
Tissue parasite distribution kinetics in TcI-JR-infected C3H/HeN mice. **A.** Representative images show parasite distribution in different organ tissue (lymph nodes—LN, gut mesenteric tissue—MS, heart—HT, spleen—SP, skeletal muscle—SKM, stomach—ST, small intestine—SI, caecum—CA and colon—CO) of a TcI-JR infected C3H mouse at 3, 6 and 30 weeks post-infection (p.i.) using *ex vivo* bioluminescence imaging. Overlaid log-scale pseudocolour heat maps are representative of bioluminescence intensity; the log-scale range is indicated in units of radiance. **B.** Box-plots show infection intensity of different organ tissue at 3 (*n* = 12 per group), 6 (*n* = 24 per group except *n* = 5 lymph nodes) and 30 (*n* = 10 per group except *n* = 5 lymph nodes) weeks p.i. Data points are expressed as fold change in bioluminescence vs. naïve controls. Limit of detection is denoted as dashed line. The horizontal line within each box indicates median and the whiskers denotes minimum and maximum values of each dataset. **C.** Scatter plots show correlation between gastrointestinal transit time and end-point parasite densities expressed as the aggregate bioluminescence radiance of the GI tract regions at 3 (*n* = 10), 6 (*n* = 12) and 30 (*n* = 10) weeks p.i.; r denotes Pearson’s correlation coefficient and p-value represents a measure of statistical significance.

We selected the TcI-JR-infected C3H mouse as the most suitable model of experimental chronic DCD. The transit time delay in these animals (Fig 1B) did not show a correlation with the overall parasite burden, which dropped by approximately two orders of magnitude from the acute peak to the level seen in the chronic phase (Fig 1A). Much of the bioluminescence signal in whole animal imaging derives from parasites in the skin [18,19], so we quantified organ-specific parasite loads using *ex vivo* imaging at 3, 6 and 30 weeks p.i. (Fig 2A). Parasitism was consistently detected in the GI tract, in foci distributed from the stomach to the rectum, being relatively more intense in the stomach and large intestine compared to the small intestine (Fig 2A and 2B). All sites exhibited significantly lower parasite loads in the chronic than acute phase (Fig 2B). There was a positive correlation between endpoint GI parasite loads and the severity of transit delay during the acute phase (3 weeks p.i.), but there were no such quantitative associations in the transition (6 weeks) or chronic (30 weeks) phases (Fig 2C). Evidence of chronic GI parasitism was found for other models displaying milder or transient transit dysfunction (C3H –TcVI-CLBR, BALB/c–TcI-JR, BALB/c–TcVI-CLBR, C3H –TcI-SN3, C3H –TcVI-Peru) but also some with normal transit times (C3H –TcI-ArePe, C3H –TcIII-Arma18) (S1 and S2 Figs). Overall the *ex vivo* imaging analyses showed that GI transit time delays (Fig 1) coincided with the persistence of *T*. *cruzi* in the GI tract (Fig 2) in the principal C3H –TcI-JR model. The relationship between transit dysfunction and gut parasitism, however, depends on additional factors because (i) there was not a consistent correlation between delay severity and infection intensity over time, and (ii) GI parasite persistence occurs apparently ubiquitously across different mouse-parasite strain combinations (Figs 2 and S2, [19,32]), yet only a subset have a functional DCD phenotype.

### Regional dissection of the transit delay phenotype reveals localisation to the colon

**Fig 3:**
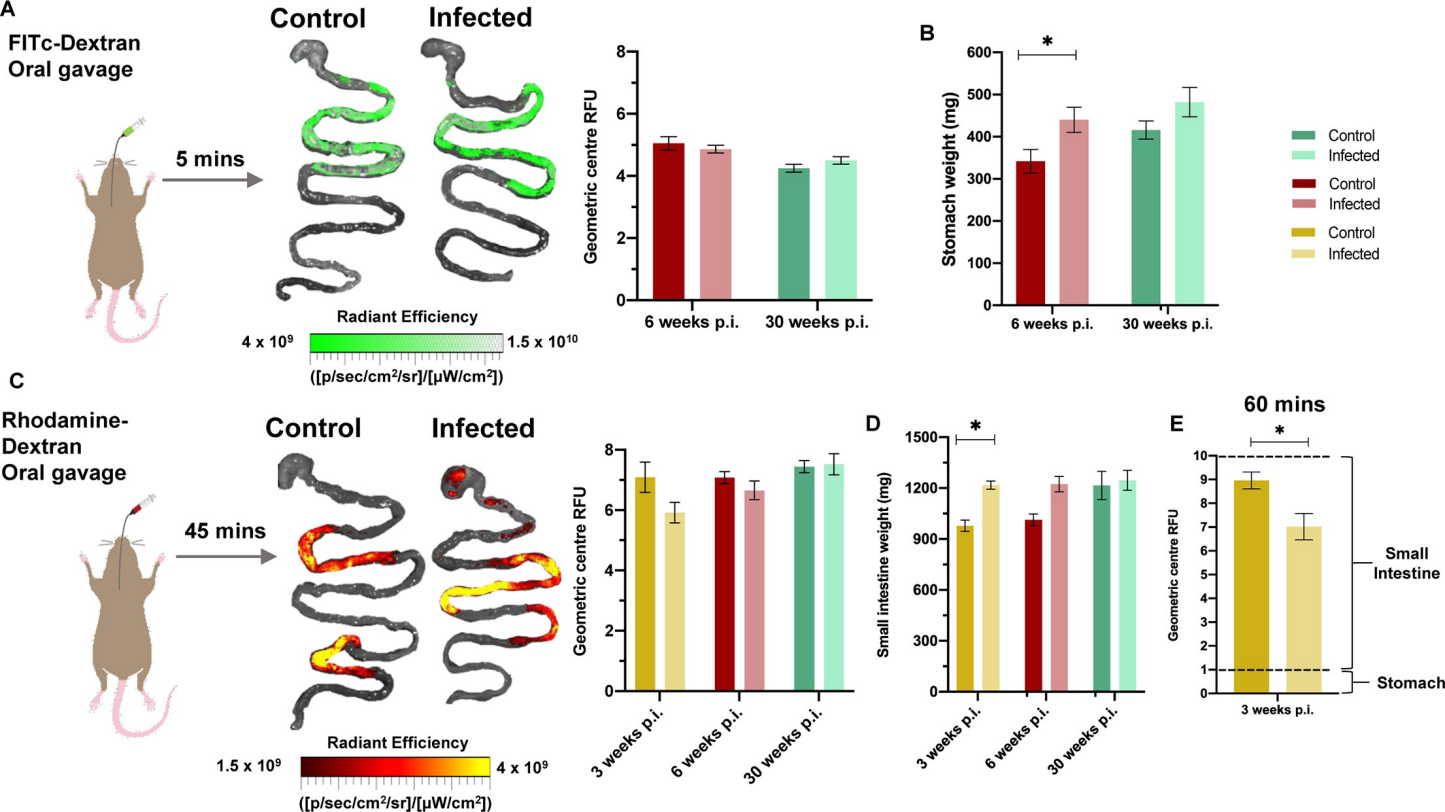
Fluorescent tracer imaging assays for stomach emptying and small intestine transit. **A.** Schematic diagram of a mouse receiving oral gavage of a green fluorescent marker, FITc-conjugated 70 kDa dextran, 5 minutes prior to termination to trace stomach emptying delay during infection. Representative images of stomach and small intestine are superimposed with traces of FITc-dextran travelling through stomach into small intestine to show transit difference between control and TcI-JR C3H/HeN infected mice. Linear-scale pseudocolour heat map shows minimum and maximum fluorescence intensity of 70-kDa FITC-dextran. Quantification of FITC-dextran fluorescence in control naïve and TcI-JR C3H/HeN is shown in the adjacent bar plot at 6 (*n* = 12 per group) and 30 (*n* = 5 per group) weeks post-infection (p.i.). Fluorescence is expressed as geometric centre which is centre mass of the marker. **B.** Bar plot shows post-mortem weights of stomach with contents at 6 (*n* = 7 per group) and 30 (*n* = 5 per group) weeks p.i. **C.** Similar schematic diagram and bar plot at 3 (*n* = 4 per group), 6 (*n* = 4 per group) and 30 (*n* = 5 per group) weeks p.i. using a red fluorescent marker, rhodamine-dextran, to target small intestine transit. Linear-scale pseudocolor heat map shows minimum and maximum fluorescence intensity of rhodamine-dextran. **D.** Small intestine weights shown in bar plot at 3 (*n* = 4 per group), 6 (*n* = 7 per group) and 30 (*n* = 5 per group) weeks p.i. **E.** Bar plot shows quantification of rhodamine-dextran fluorescence administered 60 minutes before termination of mice at 3 weeks p.i. (*n* = 4 per group). Dashed lines on bar plots show the GI segment number corresponding to the geometric centre score (0–1 = stomach, 1–10 = small intestine, proximal to distal). Data are expressed as mean ± SEM. Statistical significance was tested using unpaired two-tailed Student’s t test (*P < 0.05).

The transit time delay seen in symptomatic DCD animals was not explained by differences in body weight or intestine length (S3 Fig). This suggested a functional impairment to peristalsis, as seen in human DCD. Our next aim was to determine the digestive tract region(s) in which the transit time delay was localised. To do this we fed mice with red and green fluorescent tracers (Rhodamine- and FITC-conjugated 70 kDa dextran, respectively) at variable time intervals prior to *ex vivo* imaging. An interval of 5 minutes was used to test whether stomach emptying was delayed. No significant differences were detected in infected animals compared to controls (Fig 3A), either at 6 or 30 weeks p.i. There was a significant difference in stomach weight at 6 weeks p.i. (Fig 3B), which may indicate increased retention of matter more solid than the tracer.

To measure small intestine dysfunction, we initially analysed tracer transit after 45 minutes and observed a trend for delay in infected mice during the acute but not the chronic phase (Fig 3C). At 3 weeks p.i. there was also significantly increased organ weight (Fig 3D), so we extended analysis at this time point using an increased parasite inoculum and extended the tracer interval time to 60 minutes. Here, we observed evidence of significant small intestine transit delay (Figs 3E and S4).

**Fig 4:**
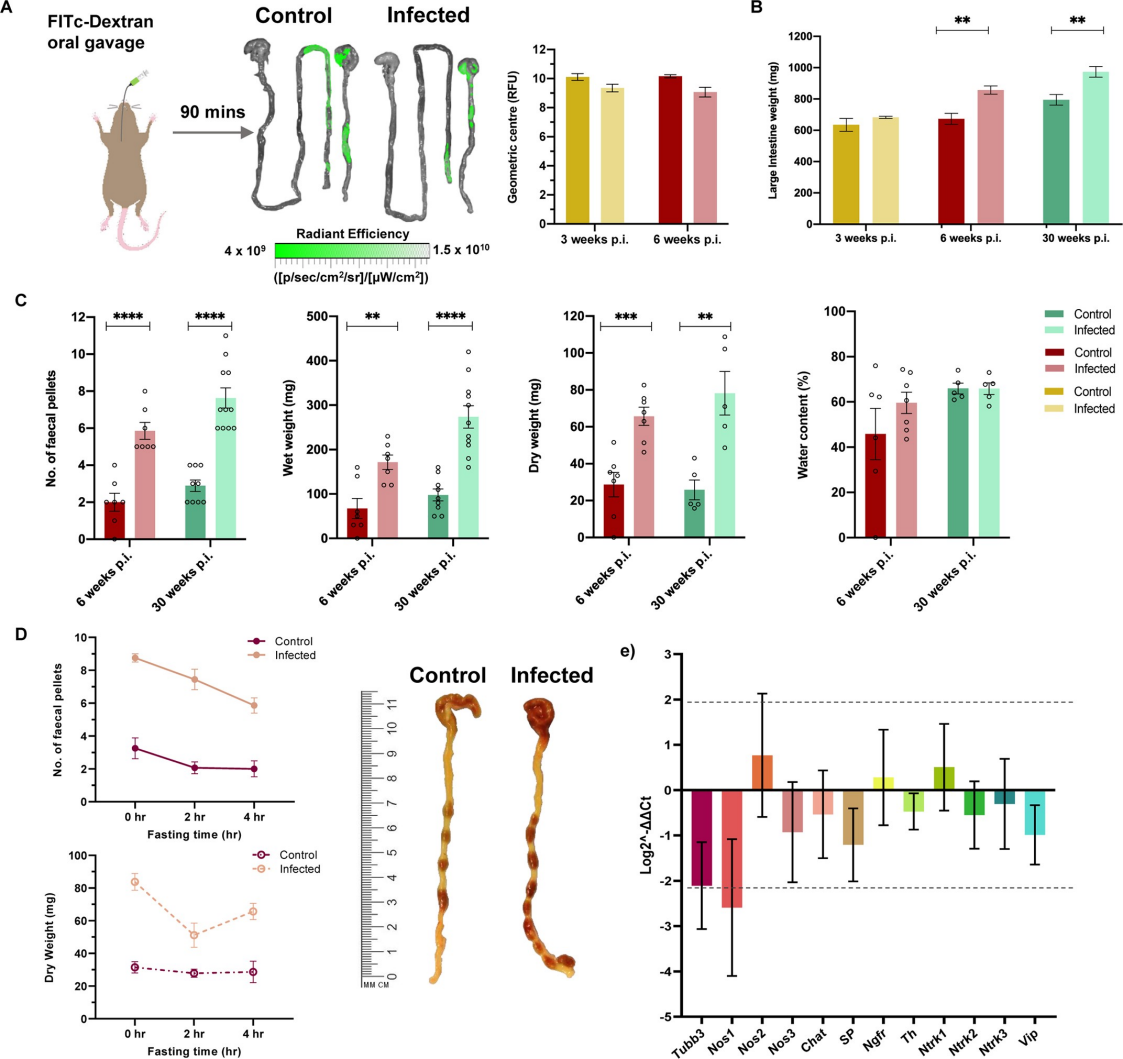
Evidence of colonic transit dysfunction in experimental digestive Chagas disease model. **A.** Schematic diagram of a mouse receiving oral gavage of a green fluorescent marker, FITC-conjugated 70 kDa dextran, 90 min prior to termination to trace large intestine transit delay during infection. Representative images of stomach, small and large intestine are superimposed with traces of FITC-dextran travelling through small into large intestine to show transit difference between control and TcI-JR C3H/HeN infected mice. Linear-scale pseudocolor heat map shows minimum and maximum fluorescence intensity of FITC-dextran. Bar plots show quantification of FITC-dextran fluorescence in the large intestine of mice at 3 (*n* = 4 per group) and 6 (*n* = 4 per group) weeks post-infection (p.i.). Fluorescence is expressed as geometric centre which is centre mass of the marker. **B.** Bar plot shows post-mortem weights of large intestine at 3 (*n* = 4 per group), 6 (*n* = 7 per group) and 30 (control *n* = 9, TcI-JR *n* = 11) weeks p.i. **C.** Faecal output analyses between control and TcI-JR C3H/HeN infected mice are expressed as faecal pellet count, wet and dry weight, and percentage of water content at 6 (*n* = 7 per group) and 30 weeks p.i. (*n* = 5–11 per group). **D.** Quantification of the effect of different fasting times on faecal output of mice: number of faecal pellets (*n* = 4–16 per group) and dry faecal weight (*n* = 4–7 per group). Images of mouse large intestine showing faecal impaction during infection at 30 weeks p.i. after 4 hours fasting compared to control. Scale bar is in cm and mm. Data are expressed as mean ± SEM. Statistical significance was tested using unpaired two-tailed Student’s t test (**P < 0.01; ***P < 0.001, **** *P* < 0.0001). **E.** RT-qPCR analysis show log2-fold change in RNA expression of neuronal markers, including pan-neuronal tubulin β-3 (*Tubb3*), neurotransmitter production (*Nos1*, *Chat*, *SP*, *Th*, *Vip*), neuronal growth factor receptors (*Ngfr*, *Ntrk1*, *Ntrk2*, *Ntrk3*), and non-neuronal NOS isoforms *Nos2* and *Nos3*. RNA was from colon tissue of C3H/HeN naïve control and TcI-JR infected mice (*n* = 5 per group, biological replicates). Data are expressed as Log2^-ΔΔCt^ ± SD. Dashed line represents mean ± 2SD based on distribution of naïve group values.

We next assessed colonic transit using a 90 minute interval after the fluorescent tracer feed. Fluorescence transit appeared similar in infected and control mice at 3 and 6 weeks p.i. (Fig 4A). Unlike the timings used to study transit delay in the upper intestinal tract (Fig 3), the method was less reliable to study the colon in isolation because substantial amounts of dye were still present in the small intestine and we could not quantify any dye that was excreted. Nevertheless, large intestine weights were significantly increased in infected mice at 6 and 30 weeks p.i. (Fig 4B) suggesting a site-specific dysfunction. We therefore employed an alternative assay in which mice were fasted for 4 hours prior to post-mortem analysis of colon lumen contents. *T*. *cruzi* infected animals showed significantly greater retention of faeces inside the colon than controls, as shown by pellet counts and both wet and dry total faecal weights, ruling out altered water absorption as an explanation (Fig 4C). The colon-localised transit delay phenotype was highly significant at 6 weeks p.i. and endured into the chronic phase, at 30 weeks p.i. (Fig 4C). By varying the fasting time (0, 2 and 4 h) we showed that this phenotype was maintained irrespective of stomach fullness and showed distal colon faecal impaction developing in *T*. *cruzi* infected mice within this timeframe (Fig 4D). The other *T*. *cruzi* strains exhibiting signs of total GI transit delay in the carmine assay (SN3, Peru, CLBR) also showed significant retention of faeces after 4 hour fasting, whereas strains with normal carmine transit times did not (S5 Fig). Thus, when GI transit dysfunction occurs in murine chronic *T*. *cruzi* infections it is predominantly localised to the colon.

To further investigate whether the observed functional constipation phenotype was accompanied by alterations at the molecular level, we used RT-qPCR to measure transcript abundance for 12 neuronal and inflammatory response genes in colon tissue from chronically infected mice (Fig 4E). Neuron-specific tubulin β-3 (*Tubb3*) and neuronal nitric oxide synthase (*Nos1*) genes were strongly downregulated by ~75% compared to naïve control mice. Expression of excitatory substance P and inhibitory vasoactive intestinal peptide (*Vip*) ENS neurotransmitters was also decreased, but to a lesser extent. No evidence of altered transcript abundance was found for markers of other enteric neuronal subtypes, tyrosine hydroxylase (*Th*) and choline acetyltransferase (*Chat*), tropomyosin receptor kinases (*Ntrk1*/2/3) or nerve growth factor receptor (*Ngfr*). Taken together, these data indicate a possible downregulation of the enteric nitrergic transmission associated with GI dysfunction in DCD mice, recapitulating observations in human Chagas megasyndromes as well as other enteric neuropathies [16,33–36].

### Chronic infection foci and enteric neuronal damage at organ and tissue micro-domain scales

**Fig 5:**
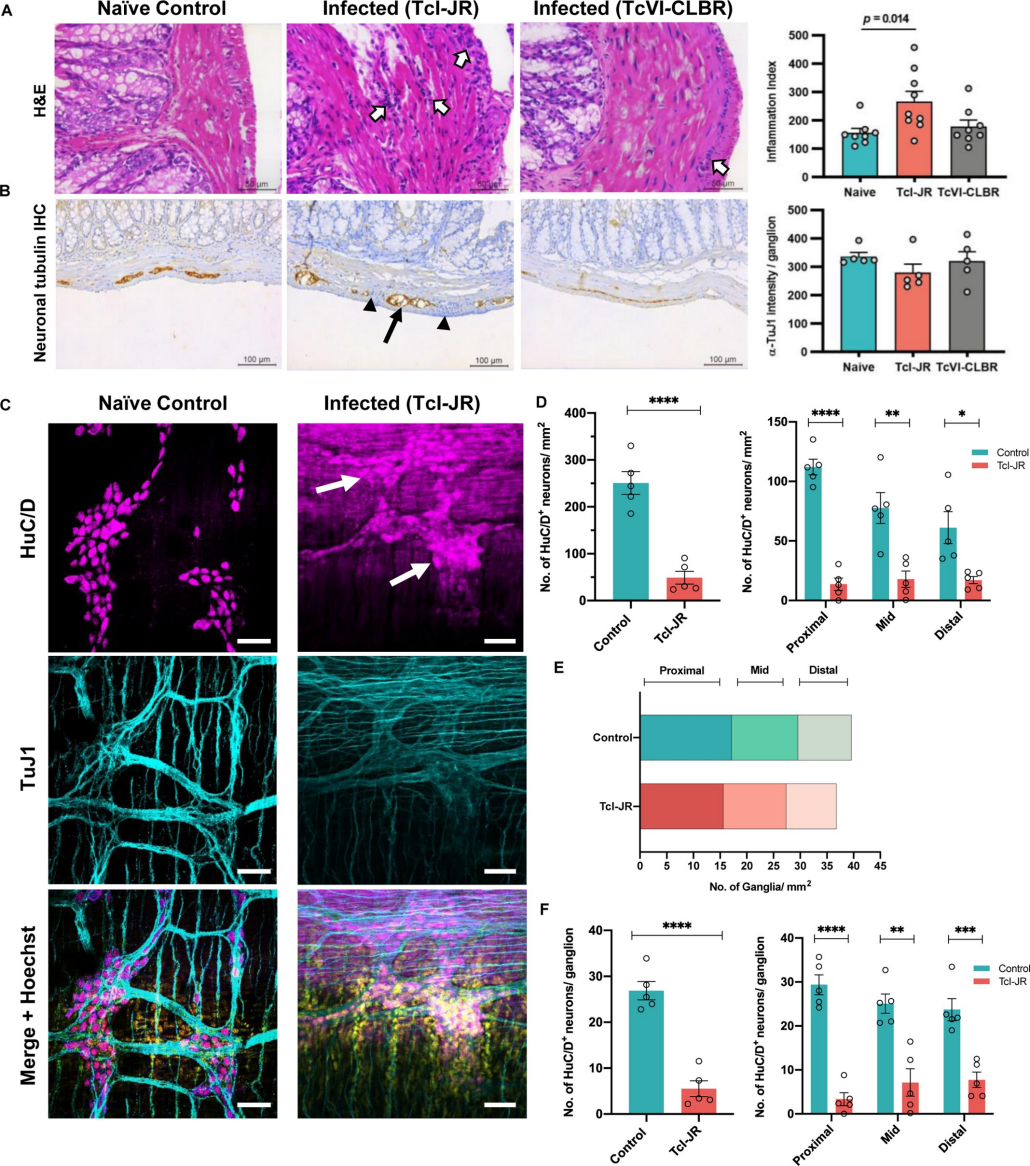
Effects of chronic *T*. *cruzi* infection on the enteric nervous system. **A.** Representative brightfield images of 5 μm thick colon sections stained with haematoxylin-eosin. Samples were cut as transverse cross-sections and images are oriented to show the mucosa to the left of the smooth muscle layers and serosa at the right edge. Inflammatory infiltrates are apparent in infected samples, visible as increased numbers of haematoxylin (purple) stained nuclei (white arrows). Images were taken at 400X magnification, scale bar  =  50 μm. Adjacent bar plot shows number of nuclei per field to quantify cellular infiltration in TcI-JR (*n* = 8), TcVI-CLBR (*n* = 10) infected mice compared to naïve controls (*n* = 8). **B.** Representative brightfield images of 5 μm thick colon sections to detect neuropathology during *T*. *cruzi* infection detected by immunohistochemistry. Samples were cut as transverse cross-sections and images are oriented to show the mucosa above the smooth muscle layers and serosa at the lower edge. Sections were labelled with a pan-neuronal antibody (TuJ1) detected as (3,3’-Diaminobenzidine, DAB) chromogen deposits (brown) and counter-stained with haematoxylin (blue), with signal predominantly localising to the myenteric neural plexus situated between the circular and longitudinal muscle layers. Black triangles indicate inflammatory foci, black arrow indicates disorganised TuJ1 distribution in a myenteric ganglion. Images were taken at 200X magnification, scale bar  =  50 μm. Adjacent bar plot shows percentage of neuronal tubulin (TuJ1) immunoreactivity in naïve control (*n* = 8), TcI-JR- (*n* = 8) and TcVI-CLBR (*n* = 9) infected mice. **C.** Representative immunofluorescent confocal images of whole-mount colon samples to show the change in anti-HuC/D stained neuronal cell bodies (magenta, top panel) and anti-Tuj1 stained neural network (cyan, middle panel) in the myenteric plexus during *T*. *cruzi* infection. Bottom panel shows merged images with DAPI nuclei stain (yellow). White arrows indicate damaged ganglionic neuronal cell bodies. Images were taken at 400X magnification, scale bar = 50 μm. **D.** Bar plots show number of HuC/D positive neuronal cell bodies per field of view in naïve control and TcI-JR infected whole colon samples (left) and from selected regions of the colon: proximal, mid and distal (right; *n* = 5 per group, all). **E.** Quantification of number of ganglia in naïve control and TcI-JR infected samples from proximal, mid and distal colon (*n* = 5 per group). **F.** Bar plots show number of HuC/D positive neuronal cell bodies per ganglion in naïve control and TcI-JR infected whole colon samples (left) and from selected regions of the colon: proximal, mid and distal (right; *n* = 5 per group, all). All data and images are obtained from matched naïve control and infected mice at 30 weeks post-infection. Data are expressed as mean ± SEM. Statistical significance was tested using unpaired two-tailed Student’s t test (*P < 0.05; **P < 0.01; ***P < 0.001, **** *P* < 0.0001).

Our next aim was to investigate disease pathogenesis in this model and commonalities with human DCD. Colon tissue from TcI-JR chronically infected mice (> 210 days p.i.) contained significant lymphocytic inflammatory infiltrates that were diffusely and focally distributed in the smooth muscle layers (Fig 5A). Immunohistochemical labelling of the nerve plexuses within the muscle layers showed that the total amount of neuron-specific tubulin (TuJ1) protein within myenteric ganglia was lower on average in infected mice, but this was not statistically significant (Fig 5B). However, there was a conspicuous spatial disorganisation of TuJ1 in a subset of ganglia, associated with the appearance of anomalous internal acellular structures in these ganglia, which were refractory to common histological dyes (Figs 5B and S6). To investigate this with greater precision, we used whole mount immunofluorescence analysis of the neuronal cell body marker HuC/D. This revealed a dramatic loss of neurons across the proximal, mid and distal colon myenteric plexus (Figs 5C and 5D). This was not a product of a reduced number of ganglia (Fig 5E), rather a highly significant reduction in neurons per ganglion (Fig 5F).

**Fig 6:**
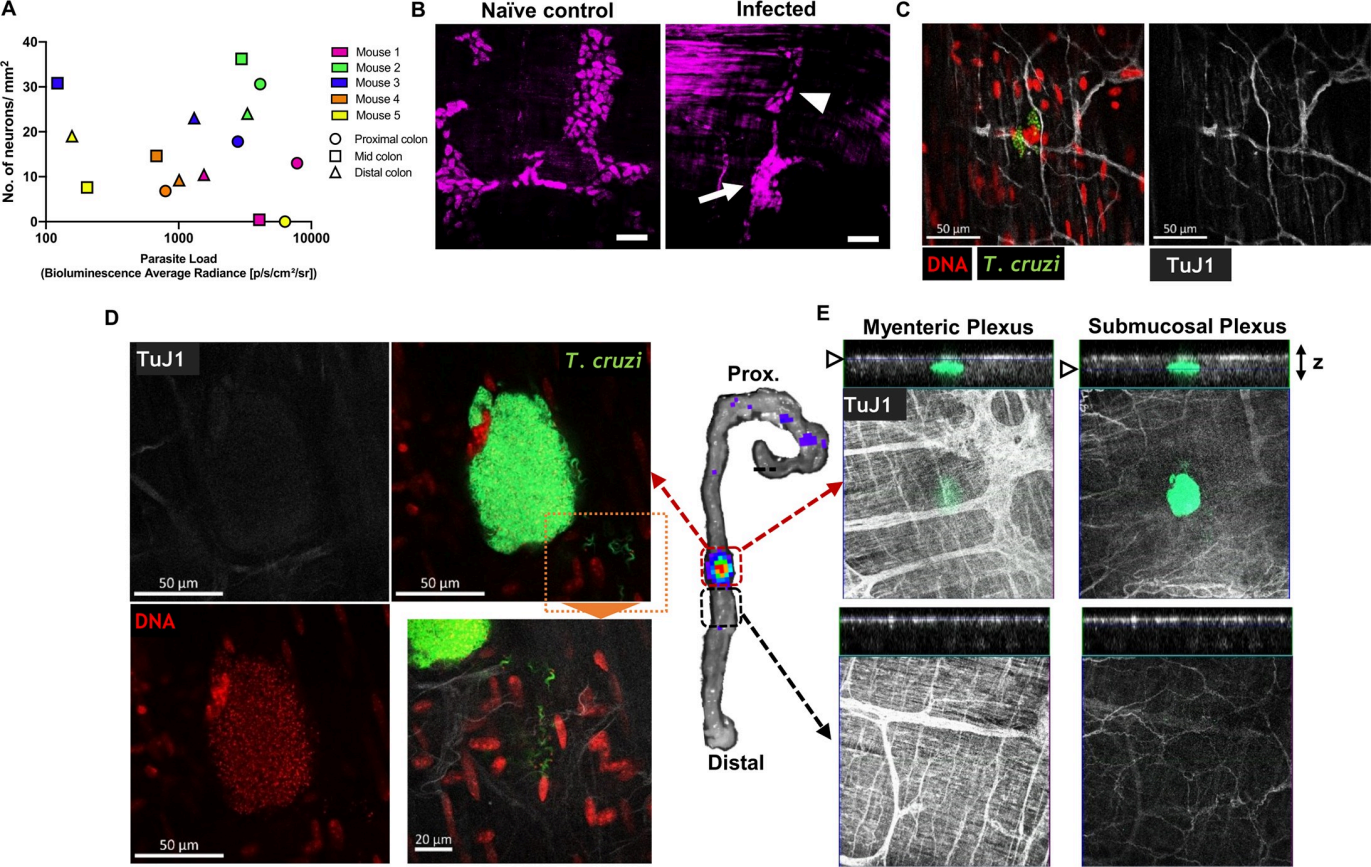
Chronic phase colonic *T*. *cruzi* infection foci and ENS ablation at the tissue micro-domain scale. **A.** Lack of correlation between end-point colon parasite loads measured by *ex vivo* bioluminescence intensity and degree of colon myenteric plexus denervation. **B–E.** Whole mount immunofluorescence analysis of colonic muscularis from C3H mice chronically infected with *T*. *cruzi*. **B.** HuC/D^+^ neuronal cell bodies in colonic myenteric ganglia. Naïve control shows normal morphology; infected mice exhibit adjacent ganglia with both intact (arrowhead) and disrupted (arrow) staining patterns. **C.** Imaging individual *T*. *cruzi* (mNeonGreen^*+*^) infected cells at early stage of parasite replication cycle adjacent to intact enteric neuron fibres (TuJ1^+^). **D–E.** Bioluminescence *ex vivo* image (centre)-guided analysis of parasitized and parasite-free tissue micro-domains. **D.** Mature parasite pseudocyst containing >1000 flagellated trypomastigotes with extracellular trypomastigotes in the local tissue parenchyma (inset) with faint neuronal (TuJ1) staining. **E.** Z-stack slices at the level of myenteric and submucosal neuronal plexuses showing highly localised loss of TuJ1 staining around the rupturing parasite pseudocyst.

A critical question for rational design of therapies for DCD is whether *T*. *cruzi* and the associated host response continues to drive ENS pathology during the chronic phase of infection. At this stage, very few colon cells are infected at any one time and parasite foci are spatiotemporally dynamic, with an intracellular lytic cycle lasting 1–2 weeks before motile trypomastigotes migrate within and between tissues [37]. Thus, any temporal association between infection and ENS damage is likely highly localised and rare at any snapshot in time. Indeed, there was no correlation between chronic endpoint parasite loads in colon regions and the level of local denervation (Fig 6A). We also observed both denervated and intact myenteric ganglia immediately adjacent to each other (Fig 6B). Using dual bioluminescent-fluorescent reporter parasites [38] we were able to visualise rare chronic infection foci at single cell resolution. In most cases, infected cells were early in the proliferative cycle, with 10–50 amastigote forms, and they were located in close proximity to intact enteric nerve fibres (Figs 6C and S7). We also captured a very rare, mature pseudocyst at the point of rupture, with thousands of intracellular parasites and trypomastigote forms escaping the site (Fig 6D). The ENS at the level of this pseudocyst was almost completely ablated, whereas the overlying and laterally adjacent ENS networks were intact (Fig 6E). Taken together, our data demonstrate there is an enduring association, at a highly localised tissue micro-domain scale, between chronic parasitism of the gut wall and ENS lesions.

## Discussion

Understanding of the mechanism of DCD pathogenesis remains rudimentary and a lack of experimental tools hampers progress. Post-mortem and biopsy studies of human DCD cases found reduced numbers of enteric neurons and glial cells, often coincident with intact parasites, *T*. *cruzi* DNA or antigen and inflammatory infiltrates [2,10–17,39,40]. These are important insights into late and terminal disease states, but they provide limited information on pathogenesis and relationships with infection load or distribution over time, which can only realistically be studied in animal models. *T*. *cruzi* infected mice do not develop advanced digestive megasyndromes resembling those in humans, but these are late stage manifestations that usually take many years to develop. Nevertheless, denervation and other features of nascent enteropathy have been described in several mouse models at the histological level [27,41–45]. Delayed transit has also been reported [44,46,47], but neither the GI region involved, nor associations with infection dynamics have been determined. In this study, we present new experimental chronic *T*. *cruzi* infection imaging models that, crucially, feature co-localised colonic parasite persistence, denervation and delayed transit as a key functional symptom of DCD.

Our data show that the transit delay seen in chronically infected mice predominantly localises to the colon. It should be noted that in humans, digestive disease affects the oesophagus at a similar frequency [40,48], but we did not study this region, mainly because of the technical challenges targeting a functional assay to the murine oesophagus [49]. Also, evidence indicates the murine oesophagus is not chronically parasitized by *T*. *cruzi*, or is below the limit of detection for imaging, which might reflect intrinsic anatomical differences between mice and humans in the upper GI tract [50]. The colon, therefore, presents better opportunities for experimental investigation of pathways connecting infection, inflammation and tissue damage. Moreover, enteric neuronal lesions and losses are the central feature in all digestive forms of Chagas disease [51], so the focus on the colon in this model is likely to generate data that can usefully be extrapolated, with due caution, to other GI regions including the oesophagus. There are also wider reasons to be optimistic about the translational value of these murine DCD models. For example, they perform well in predicting the efficacy of anti-parasitic drugs in clinical trials, in terms of both cure of infection and impact on cardiac tissue pathology [52].

The digestive manifestations of Chagas disease predominantly occur in parts of South America where the majority of human *T*. *cruzi* infections are caused by strains from lineages TcII, TcV and TcVI. Conversely, DCD is apparently far rarer north of the Amazon basin, where most human infections involve TcI. While this geographical association may well be circumstantial, being confounded by eco-epidemiological factors and human population genetic variability [53,54], it is still noteworthy that, in mice, a) GI transit delays were strain and not lineage-specific; and b) the most robust phenotype was caused by a TcI strain. There have been reports of TcI infections in humans with symptoms of digestive disease in Colombia [55,56], and they probably also occur in Venezuela [51] and Mexico [57]. It may be that disease presentation is milder in TcI-endemic areas without progression to megasyndromes and is less likely to be diagnosed. It should also be acknowledged that *in vitro* culture adaptation, genetic manipulation and clonal selection may be factors affecting the strain-specific phenotypes observed in our work. Nevertheless, our data still enable us to conclude that both host and parasite genetics contribute to murine DCD susceptibility. With respect to the host, evidence to date indicates that the digestive tract is probably a ubiquitous reservoir of chronic infection in mice. Disease severity was higher in C3H/HeN than BALB/c mice, a finding previously observed for murine cardiac CD [19], and is consistent with the heterogeneous clinical outcomes observed in humans [1].

A key conclusion is that gut parasitism alone is not sufficient as an explanation for DCD development, though it is likely to be a pre-requisite. The quantity and quality of the host’s immune and tissue repair responses are likely to be central to disease resistance and susceptibility, yet understanding of gut-specific immunity in Chagas disease is mainly limited to descriptions of the general composition of inflammatory infiltrates, which are rich in T and NK cells [2,13,16]. Other outstanding questions include whether the patchiness of ENS damage is explained by the stochastic distribution of parasites, or because particular subsets of ganglia or neurons differ in susceptibility [58], and if so, why? We focussed on analysis of neurons in the myenteric plexus, but it will be important to explore other ENS components, including potential regulatory or neuroprotective functions of enteric glial cells [2,16,59] and broader factors known to influence neuro-immune interactions in the gut, such as host metabolism [60] and microbiota [61].

Analysis of the kinetics of disease development in our main DCD model (TcI-JR infected C3H mice) showed that the intensity of gut parasitism was somewhat correlated with the degree of transit delay around the peak of the acute phase of infection, but this correlation disappeared after the transition to the chronic phase. Transit assays and imaging were done on different days, so it may be that in the acute phase, where parasite loads are very high and more evenly distributed, snapshot parasite load measurements are more representative of wider time frames than they are in the chronic phase, when parasites are more focal and levels fluctuate substantially within and between individual hosts over time [18]. Also, the main effector causing collateral damage to the ENS in the acute phase is likely to be excessive NO production via iNOS [9], however, the later transition and chronic inflammatory environments are different situations, in that they promote an equilibrium between parasite replication and suppression by host adaptive immunity [62]. Neuro-immune networks in the gut are also highly diverse [63], so additional mechanisms of pathogenesis may come into play during the chronic phase. Investigation of parasite virulence and variability in the host immune response will therefore be required to gain further insight into the determinants of susceptibility and resistance.

In summary, by combining live parasite imaging and gut tracer analyses, we found enduring associations between infection of the colon and local transit impairment at >6 months post-infection, and moreover at the tissue micro-domain scale between single infected cells and ENS lesions. Our results challenge the theory that DCD is a result of collateral damage to the ENS, resulting specifically from the acute inflammatory response against *T*. *cruzi* [7,64]. They support the interpretation that the presence of *T*. *cruzi* and inflammatory infiltrates in GI tissues of human DCD patients reflects a long-term association between parasite persistence and disease development [11,13,15]. However, this still does not prove a causal relationship between local infection and pathogenesis, nor the kinetics of the process; this will require elimination of *T*. *cruzi* at defined time points through anti-parasitic chemotherapy, for example using benznidazole or nifurtimox. More importantly, such experiments will help to predict whether treatment of chronic infections might have the potential to prevent or alleviate DCD in humans.

## Materials and methods

### Ethics statement

All *in vivo* experiments were performed in accordance with UK Home Office regulations under the Animal Scientific Procedure Act (ASPA) 1986, within the framework of project license 70/8207 or P9AEE04E, and were approved by LSHTM Animal Welfare Ethical Review Board.

### Parasites and infections

Transgenic clones of *T*. *cruzi* TcI-JR and TcVI-CLBR constitutively expressing the red-shifted firefly luciferase variant *PPy*RE9h [28], alone or fused to mNeonGreen, were described previously [19,38]. Equivalent clones for other *T*. *cruzi* strains were generated by transfection of the DNA construct pTRIX2-RE9h (TcI-C8, TcI-X10/4, TcIII-Arma18, TcVI-Peru), or by cas9-mediated replacement of the LucRE9h gene with dual reporters, namely LucRE9h::Neon (TcVI-CLBR, TcII-Pot7a, TcIV-X10610) and LucRE9h::mScarlet (TcI-ArePe, TcI-SN3), using the T7 RNA polymerase/cas9 system [38]. *In vitro* epimastigotes were cultivated in supplemented RPMI-1640 medium at 28°C with 150 μg ml^-1^ G418 or hygromycin B, 5 μg ml^-1^ puromycin or 10 μg ml^-1^ blasticidin as appropriate. Metacyclic trypomastigotes (MTs) from stationary phase cultures were used to infect MA104 monkey kidney epithelial cell monolayers in MEM media + 5% FBS at 37°C and 5% CO_2_. Tissue culture trypomastigotes (TCTs) were obtained from the supernatant of infected cells after 5 to 21 days, depending on the parasite strain.

### Animals and infections

Female BALB/c and C3H/HeN mice, postnatal days 42–56, were purchased from Charles River (UK). Female CB17 SCID mice were bred in-house. All mice were housed on a 12 h light/dark cycle, with food and water available *ad libitum* unless otherwise stated. Mice were maintained under specific pathogen-free conditions in individually ventilated cages.

SCID mice were infected with up to 5 × 10^5^ *in vitro*-derived TCTs in 0.2 ml PBS via i.p. injection. All infected SCID mice developed fulminant infections and were euthanised at or before humane end-points. Blood trypomastigotes (BTs) were derived from parasitaemic SCID mouse blood directly or after enrichment, achieved by allowing blood samples to settle for 1 h at 37° C. BALB/c and C3H mice were infected by i.p injection of 10^3^ or 10^4^ BTs or TCTs depending on the experiment.

At experimental end-points, mice were sacrificed by ex-sanguination under terminal anaesthesia (Euthatal/Dolethal 60 mg kg^−1^, i.p.) or by cervical dislocation. Organs and tissues of interest were excised, imaged (see below) and either snap-frozen, fixed in 10% Glyo-Fixx (Shandon) or transferred to ice-cold DMEM media. The weight of organs and tissues of interest were recorded.

### Total GI transit time assay

Mice were gavaged p.o. with 200 μl of 6% w/v Carmine red dye solution in 0.5% methyl cellulose mixed in distilled water and returned to their home cage. After 75 min, mice were individually separated into containers and the time of excretion of the first red faecal pellet was recorded. A maximum assay cut-off time of 4 h was implemented. Total intestinal transit time was calculated as the time taken from gavage to output of the first red pellet.

### *In vivo* bioluminescence imaging

Mice were injected with 150 mg kg^−1^ d-luciferin i.p., then anaesthetised using 2.5% (v/v) gaseous isoflurane in oxygen. After 10–20 min, bioluminescence imaging was performed using an IVIS Lumina II or Spectrum system (PerkinElmer), with acquisition time and binning adjusted according to signal intensity. Mice were revived and returned to cages after imaging. To estimate parasite burden in live mice, regions of interest (ROIs) were drawn to quantify bioluminescence expressed as total flux (photons/second) [18,19]. The detection threshold was determined using uninfected control mice. All bioluminescence data were analysed using LivingImage v4.7.3.

### *Ex vivo* bioluminescence imaging

Mice were injected with 150 mg kg^-1^ d-luciferin i.p. 5–7 min before euthanasia. Trans-cardiac perfusion was performed with 10 ml of 0.3 mg ml^-1^ d-luciferin in PBS. Tissues and organs of interest (typically lymph nodes, heart, spleen, skeletal muscle, GI tract and associated mesenteries) were collected and soaked in PBS containing 0.3 mg ml^-1^ d-luciferin. Bioluminescence imaging was performed as above. To quantify parasite load as a measure of infection intensity, bioluminescence was calculated by outlining ROIs on each sample and expressed as radiance (photons second^−1^ cm^−2^ sr^−1^). Fold change in radiance was determined by comparing samples from infected mice with the equivalent tissues from uninfected, age-matched control mice. To determine the detection threshold, fold change in radiance of empty ROIs on images from infected mice was compared with matching empty ROIs on images from uninfected controls [18].

### Fluorescent tracer transit assay

Mice were fasted (or not) for 2 or 4 h before euthanasia. They were administered FITC-conjugated 70 kDa dextran (100 μl, 5 mg ml^-1^ d.H_2_O) or Rhodamine-conjugated 70 kDa dextran (100 μl, 10 mg ml^-1^ d.H_2_O) by oral gavage 5, 45 or 90 min before euthanasia to target the stomach, small or large intestine transit, respectively. As an extension of the *ex vivo* bioluminescence necropsy (see above), fluorescence images were obtained using excitation filters set at 465/535 nm and emission filters at 502/583 nm for FITC/Rhodamine (f-stop: 16, exposure: 2 s). The relative fluorescence of the tracers was measured from the images by drawing ROIs using LivingImage 4.7.3 software. The GI tract images starting from the stomach to the colon were cut digitally in 15 equal segments and the centre of mass (geometric centre) of the signals were determined. The geometric centre was calculated using the following equation, GC = ∑ (% of total fluorescent signal per segment * segment number) / 100) [65].

### Faecal analyses

The colon tissue was separated and cleaned externally with PBS. The faecal pellets were gently removed from the lumen of the colon, counted and the combined wet weight was recorded. The faecal pellets were collected into a 12-well plate and left to dry in a laminar flow cabinet overnight. The dry weight was then recorded and the percent water content was estimated as the difference between wet and dry weights.

### Histopathology and Immunohistochemistry

Paraffin-embedded, fixed tissue blocks were prepared and 3–5 μm sections were stained with haematoxylin and eosin as described [19,32]. For tubulin β-3 immunohistochemistry, sections were subjected to heat-induced epitope retrieval by incubation in 10 mM sodium citrate, 0.05% Tween20 for 30 min at 95°C then cooled and rinsed in distilled water. Sections were blocked with 10% sheep serum and 1% BSA in TBS for 30 min then incubated at 4°C overnight with 1 μg ml^-1^ rabbit polyclonal anti- tubulin β-3 IgG (Biolegend) and 1% BSA in TBS. Sections were then washed with 0.025% Triton X-100 in TBS and endogenous peroxidase activity was quenched with 3% H_2_O_2_ for 30 min. Bound primary antibody was labelled with excess volume of HRP polymer anti-rabbit IgG reagent (Vector Labs) with 1% BSA in TBS for 30 min. Slides were then washed as previously and incubated with DAB (Thermo) for 5 min. Sections were counterstained with haematoxylin and mounted with DPX.

Images were acquired using a Leica DFC295 camera attached to a Leica DM3000 microscope. For analysis of inflammation, nuclei were counted automatically using the Leica Application Suite V4.5 software (Leica). DAB intensity was analysed as integrated density in ImageJ.

### Immunofluorescence analysis

Colon tissues were transferred into ice-cold DMEM after necropsy. Tissues were cut open along the mesentery line, rinsed with PBS, then stretched and pinned on Sylgard 184 plates. The mucosal layer was peeled away using forceps under a dissection microscope and the remaining muscularis wall tissue was fixed in paraformaldehyde (4% w/v in PBS) for 45 min at room temperature. Tissues were washed with PBS for up to 45 min at room temperature and permeabilised with PBS containing 1% Triton X-100 for 2 h, followed by blocking for 1 h (10% sheep serum in PBS containing 1% Triton X-100). Tissues were incubated with primary antibodies (mouse anti-HuC/D IgG clone 16A11 at 1:200 [Thermofisher], rabbit polyclonal anti-tubulin β-3 IgG at 1:500 [Biolegend]) in PBS containing 1% Triton X-100 for 48 h at 4°C. Tissues were washed with PBS, then incubated with secondary IgG (goat anti-mouse AlexaFluor-546, goat anti-rabbit AlexaFluor-633, both 1:500, ThermoFisher) in PBS containing 1% Triton X-100 for 2 h and counterstained with Hoechst 33342 (1:10 000) at room temperature. To assess antibody specificity, control tissues were incubated without the primary antibody. Tissues were mounted on glass slides using FluorSave mounting medium (Merck).

Whole mounts were examined and imaged with a LSM880 confocal microscope using a 40X objective (Zeiss, Germany). Images were captured as Z-stack scans of 21 digital slices with an interval of 1 μm optical thickness. Five Z-stacks were acquired per region (proximal, mid and distal colon), per animal. Cell counts were performed on Z-stacks after compression into a composite image using the cell counter plug-in of FIJI software. Neuronal density was calculated as the number of HuC/D^+^ neuron cell bodies per field of view. HuC/D signal was associated with high background outside ganglia in samples from infected mice, attributed to binding of the secondary anti-mouse IgG to endogenous IgG, so ENS-specific analysis was aided by anti-TuJ1 co-labelling and assessment of soma morphology. The number of intact ganglia in each myenteric plexus image was also counted, along with number of HuC/D^+^ neurons per ganglia.

### RT-qPCR

Colon tissue samples were snap frozen on dry ice and stored at −70°C. For RNA extraction, samples were thawed and homogenised in 1 ml Trizol (Invitrogen) per 30–50 mg tissue using a Precellys 24 homogeniser (Bertin). To each sample, 200 μl of chloroform was added and mixed by vortex after which the phases were separated by centrifugation at 13,000 g at 4°C. RNA was extracted from the aqueous phase using the RNeasy Mini Kit (Qiagen) with on-column DNAse digestion as per manufacturer’s protocol. The quantity and quality of RNA was assessed using Qubit Fluorimeter (Thermofisher). cDNA was synthesised from 1 μg of total RNA using Superscript IV VILO mastermix (Invitrogen), as per manufacturer’s protocol, in reaction volumes of 20 μl. A final cDNA volume of 100 μl was made by adding RNase-free DEPC water (1:5 dilution) and stored at −20°C until further use. qPCR reactions contained 4 μl of cDNA (1:50 dilution) and 200 nM of each primer (S1 Table) and QuantiTect SYBR Green PCR master mix (Qiagen) or SensiFAST SYBR Hi-ROX kit (Bioline). Reactions were run using Applied Biosystems 7500 fast RT-PCR machine (Thermofisher) as per manufacturer’s protocol. Data were analysed by the ΔΔCt method [66] using murine *Gapdh* as the endogenous control gene.

### Statistics

Individual animals were used as the unit of analysis. No blinding or randomisation protocols were used. Statistical differences between groups were evaluated using unpaired two-tailed Student’s t-test or one-way ANOVA with Tukey’s post-hoc correction for multiple comparisons. Pearson correlation analyses was used to evaluate relationships between variables. These tests were performed in GraphPad Prism v.8 or R v3.6.3. Differences of *p* < 0.05 were considered significant.

## Supporting information

S1 Fig Gastrointestinal transit dysfunction screen in mice infected with different strains of *T*. *cruzi*. Data are gastrointestinal (GI) transit times at indicated weeks post-infection (p.i.) for C3H/HeN mice in the following infection groups: naive control (*n* = 4–6), TcI-ArePe (*n* = 1–4), TcI-SN3 (*n* = 4), TcI-SylvioX10/4 (*n* = 4) and TcI-C8 (*n* = 4), TcII-Pot7a (*n* = 4), TcIII-Arma18 (*n* = 3–4), TcIV-X10610 (*n* = 4) and TcVI-Peru (*n* = 3).
(TIF)

S2 Fig *Ex vivo* analysis of parasite tissue distributions for TcI-SN3, TcI-ArePe, TcIII-Arma18 and TcVI-Peru strains of *T*. *cruzi*. **A.** Bioluminescence signal intensity (blue, low, to red, high) in tissue samples from chronically infected C3H/HeN mice. Samples, from top left to right, lung, heart, genito-urinary system, liver, GI mesentery, peritoneum, skeletal muscle, visceral adipose, large intestine, small intestine, stomach and oesophagus. **B.** Frequency of parasite detection in the indicated organs/tissues. TcI-SN3 *n* = 4, TcI-ArePe *n* = 4, TcIII-Arma18 *n* = 3, TcVI-Peru *n* = 2.
(TIF)

S3 Fig Anatomical measures of gastrointestinal *T*. *cruzi* infection mouse models. **A.** Body weights of naïve control (*n* = 3–10), TcI-JR (*n* = 5–22) and TcVI-CLBR (*n* = 5–20) infected C3H/HeN (left) and BALB/c mice (right) vs. days post-infection (p.i.). **B.** Bar plots show length of small intestine and colon of control and TcI-JR C3H/HeN mice at 3 (*n* = 24 per group), 6 (*n* = 27 per group) and 30 (*n* = 5 per group) weeks p.i. Data are expressed as mean ± SEM.
(TIF)

S4 Fig Fluorescent tracer imaging assay for gastrointestinal (GI) transit in high inoculum acute *T*. *cruzi* infection. Schematic diagram of a mouse receiving oral gavage of a green fluorescent marker, FITC-conjugated 70 kDa dextran, 60 or 120 or 180 minutes prior to termination to trace localised GI transit delay during acute infection. Quantification of FITC-dextran fluorescence in different parts of the GI tract (SI 1 –SI 10: small intestine scored into 10 equal sections, proximal to distal) of naïve control and TcI-JR C3H/HeN (*n* = 4 per group) mice at 3 weeks post-infection. All mice in this experiment were infected with a high inoculum of TcI-JR parasites (10^4^). Data are expressed as mean ± SEM.
(TIF)

S5 Fig Comparison of colonic transit dysfunction in different models of experimental digestive Chagas disease. Faecal output analyses are expressed as faecal pellet count, wet and dry pellet weight at 30 weeks post-infection (p.i.) in the following groups: naive control (*n* = 7–11), TcI-JR (*n* = 5–11), TcI-SN3 (*n* = 4), TcI-ArePe (*n* = 1), TcIII-Arma18 (*n* = 3), TcVI-Peru (*n* = 3) and TcVI-CLBR (*n* = 4–5).
(TIF)

S6 Fig Myenteric neuronal plexus lesions in *T*. *cruzi* infection. Colon tissue samples from C3H mice with chronic TcI-JR infections were cut as transverse cross-sections and images are oriented to show the mucosa above the smooth muscle layers and serosa at the lower edge. Acellular structures (arrows) within myenteric plexus ganglia that are refractory to staining by the indicated method. 400X magnification.
(TIF)

S7 Fig Chronic phase colonic *T*. *cruzi* infection foci of TcI-SN3 parasites in the ENS. Representative immunofluorescent z-stack confocal images of whole-mount colonic muscularis from C3H mice chronically infected with fluorescent TcI-SN3 (mScarlet^+^) parasites. Image shows the localisation of TcI-SN3 parasites (red) in the submucosal layer of the ENS stained with anti-TuJ1 (white). DAPI (cyan) shows DNA and white circle indicates DNA of the parasite nest. Images were taken at 400X magnification, scale bar = 50 μm.
(TIF)

S1 Table Quantitative PCR target gene and primer information. (DOCX)
