# Supplementary figures and images for: Local association of Trypanosoma cruzi chronic infection foci and enteric neuropathic lesions at the tissue micro-domain scale
Source: PLoS Pathog. 2021 Aug 23;17(8):e1009864. doi: 10.1371/journal.ppat.1009864 (PMC8412264; doi:10.1371/journal.ppat.1009864)

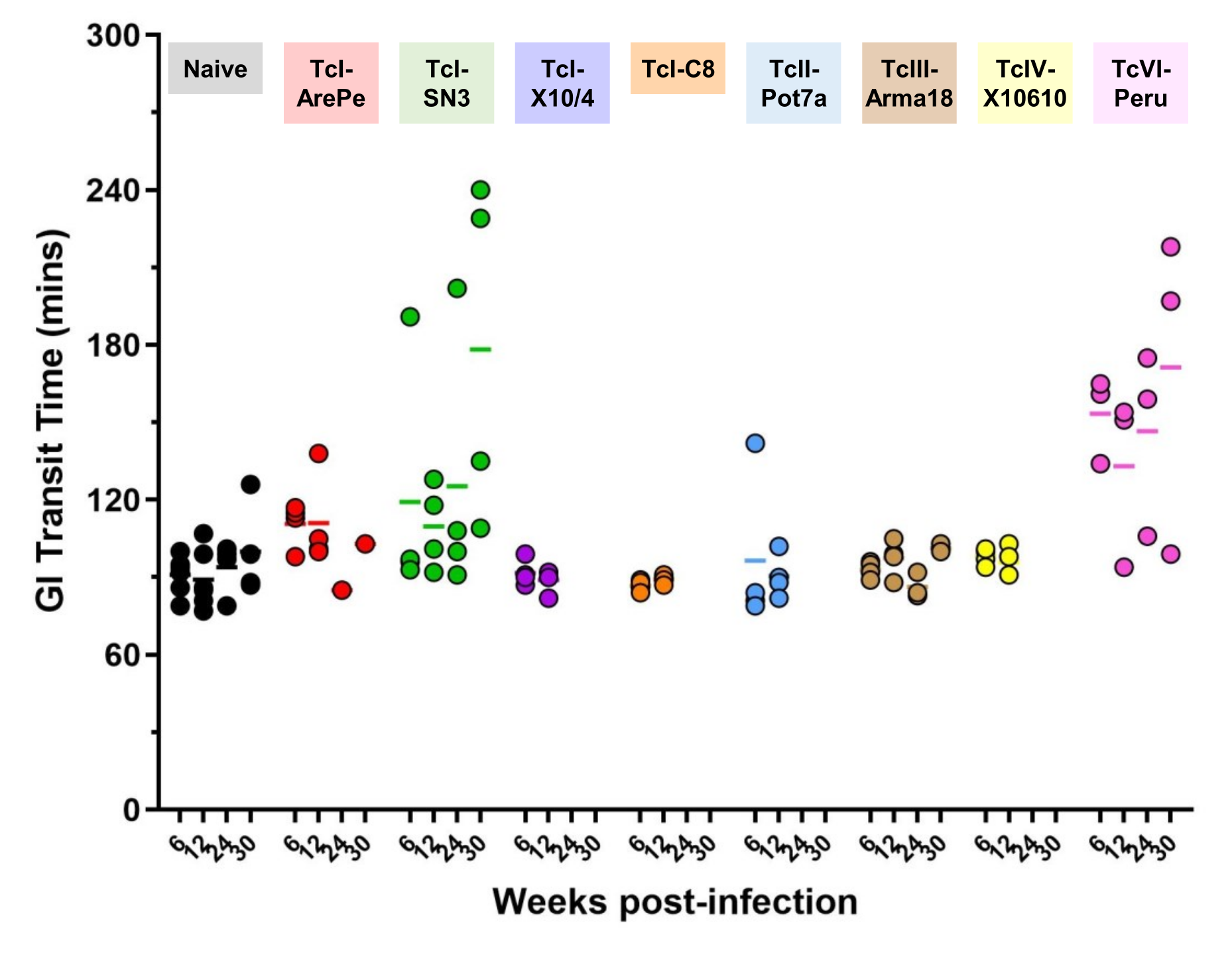

Supplement: S1 Fig — Data are gastrointestinal (GI) transit times at indicated weeks post-infection (p.i.) for C3H/HeN mice in the following infection groups: naive control (n = 4–6), TcI-ArePe (n = 1–4), TcI-SN3 (n = 4), TcI-SylvioX10/4 (n = 4) and TcI-C8 (n = 4), TcII-Pot7a (n = 4), TcIII-Arma18 (n = 3–4), TcIV-X10610 (n = 4) and TcVI-Peru (n = 3). (TIF) [file ppat.1009864.s001.tif]

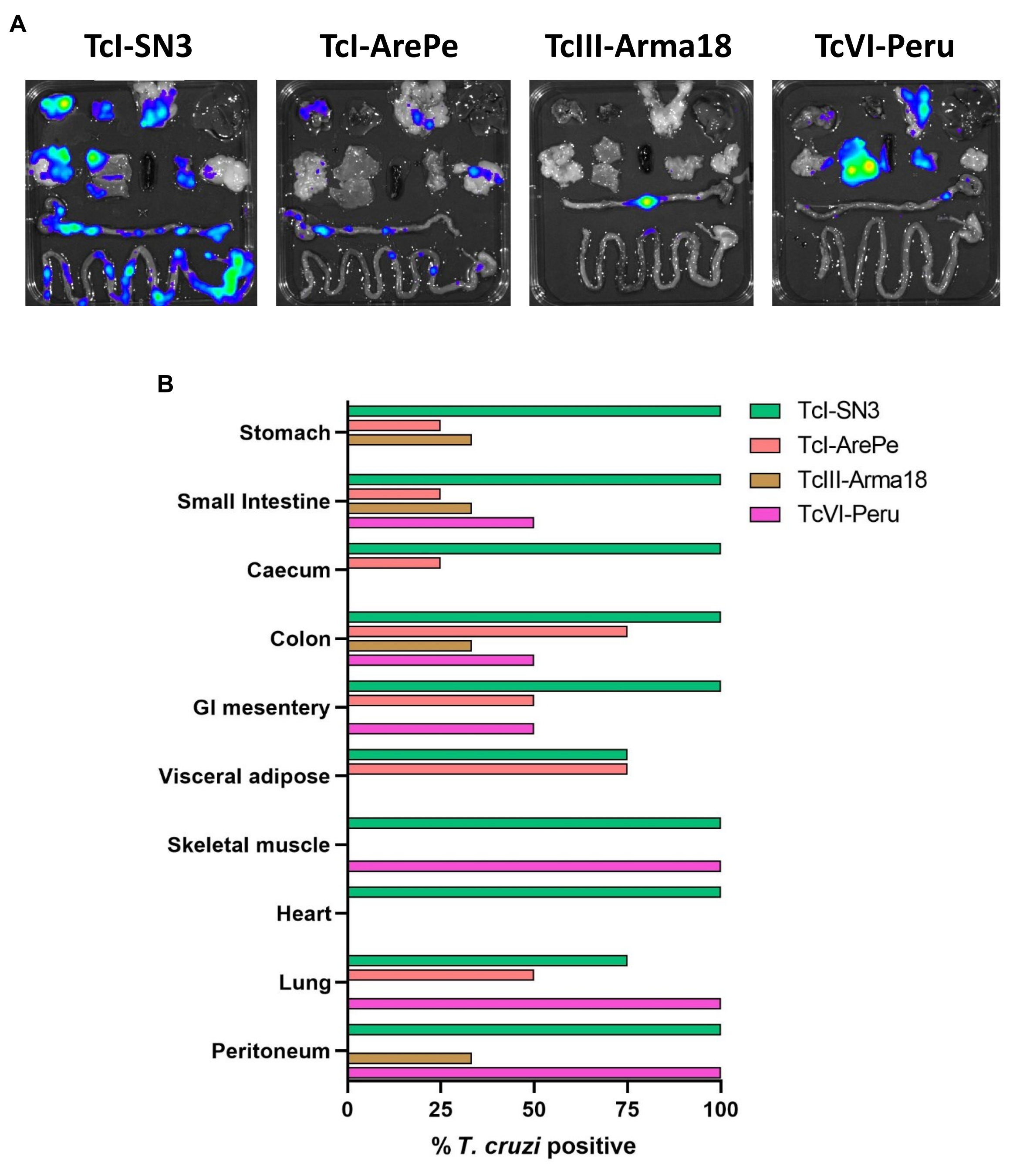

Supplement: S2 Fig — A. Bioluminescence signal intensity (blue, low, to red, high) in tissue samples from chronically infected C3H/HeN mice. Samples, from top left to right, lung, heart, genito-urinary system, liver, GI mesentery, peritoneum, skeletal muscle, visceral adipose, large intestine, small intestine, stomach and oesophagus. B. Frequency of parasite detection in the indicated organs/tissues. TcI-SN3 n = 4, TcI-ArePe n = 4, TcIII-Arma18 n = 3, TcVI-Peru n = 2. (TIF) [file ppat.1009864.s002.tif]

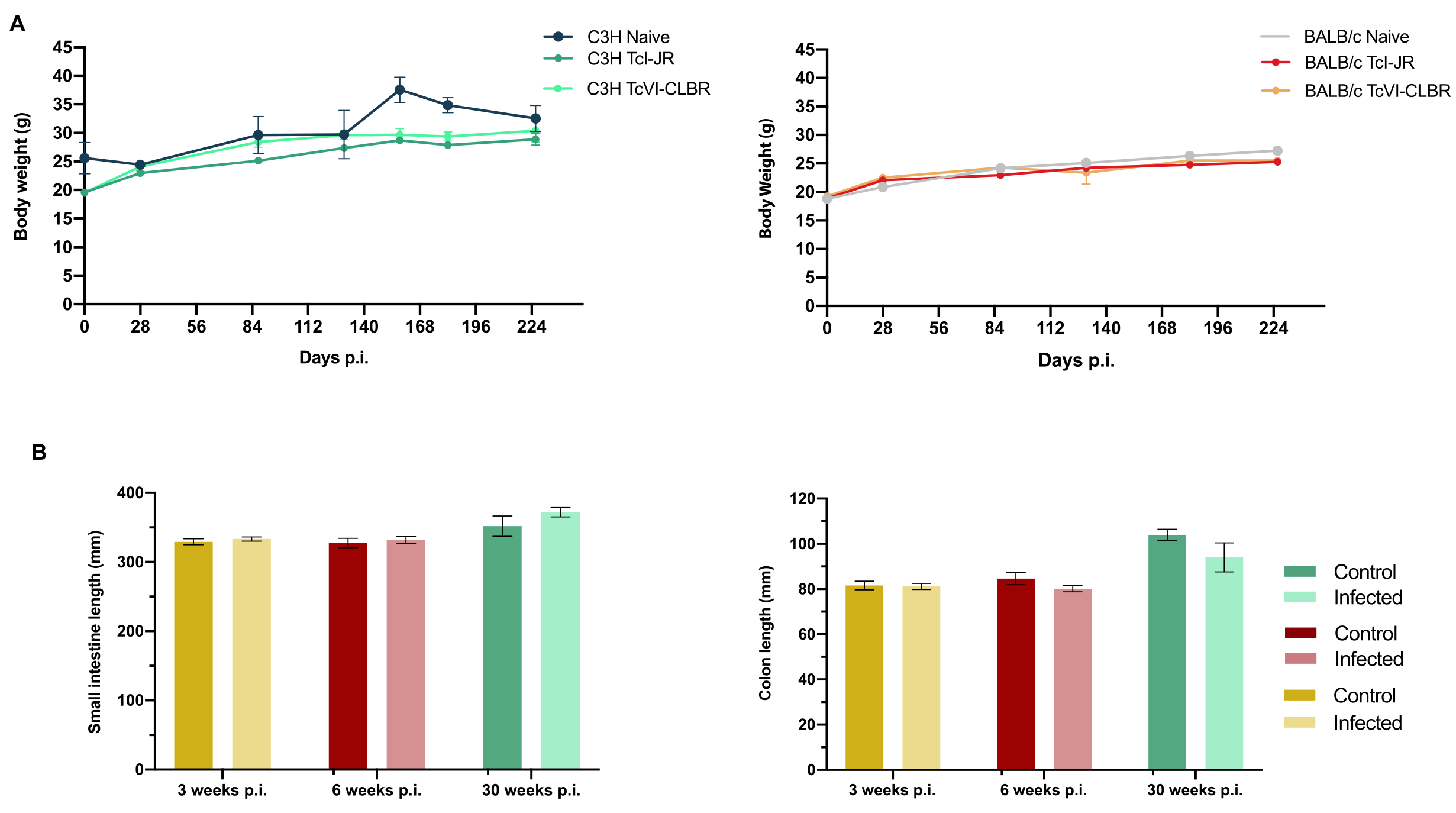

Supplement: S3 Fig — A. Body weights of naïve control (n = 3–10), TcI-JR (n = 5–22) and TcVI-CLBR (n = 5–20) infected C3H/HeN (left) and BALB/c mice (right) vs. days post-infection (p.i.). B. Bar plots show length of small intestine and colon of control and TcI-JR C3H/HeN mice at 3 (n = 24 per group), 6 (n = 27 per group) and 30 (n = 5 per group) weeks p.i. Data are expressed as mean ± SEM. (TIF) [file ppat.1009864.s003.tif]

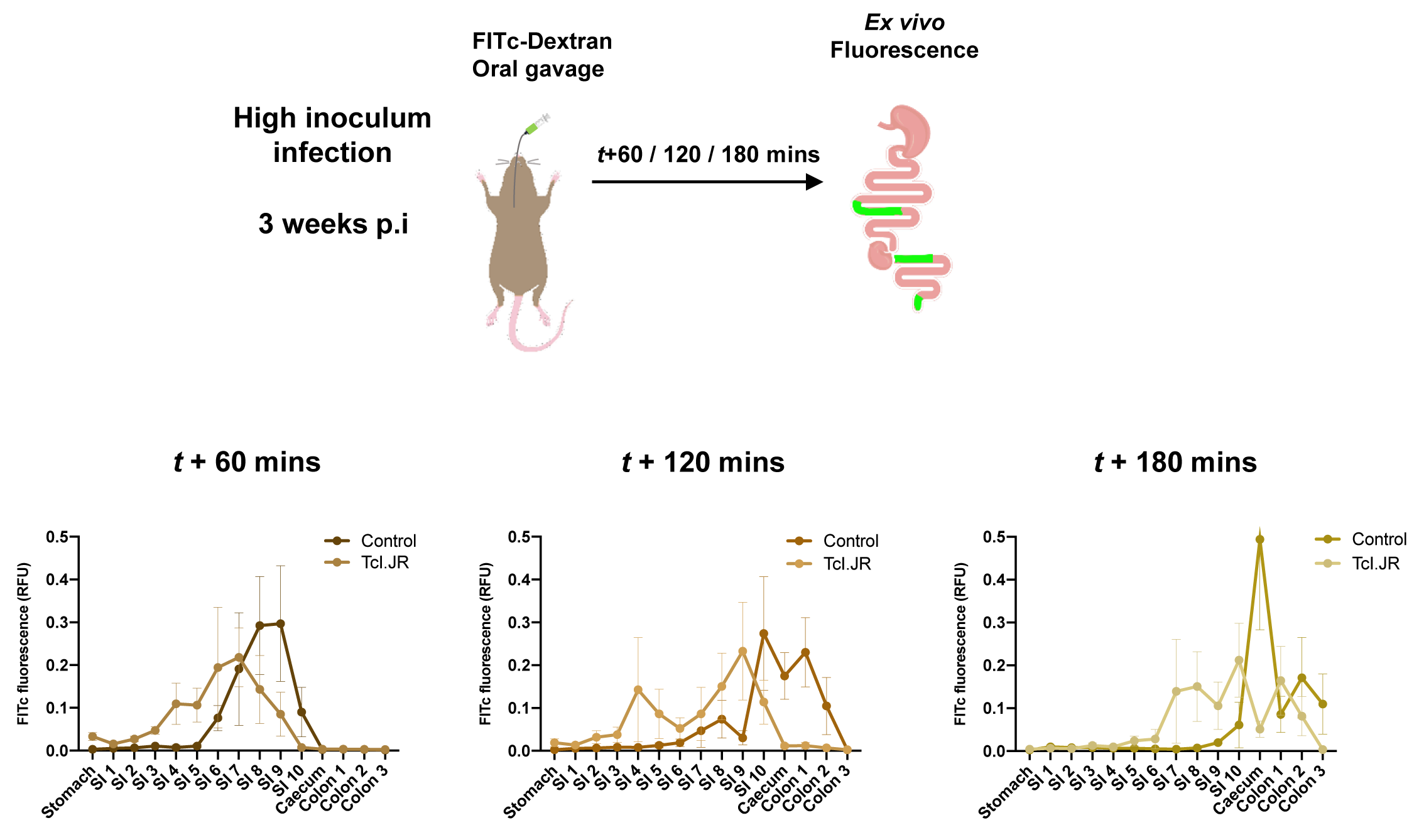

Supplement: S4 Fig — Schematic diagram of a mouse receiving oral gavage of a green fluorescent marker, FITC-conjugated 70 kDa dextran, 60 or 120 or 180 minutes prior to termination to trace localised GI transit delay during acute infection. Quantification of FITC-dextran fluorescence in different parts of the GI tract (SI 1 –SI 10: small intestine scored into 10 equal sections, proximal to distal) of naïve control and TcI-JR C3H/HeN (n = 4 per group) mice at 3 weeks post-infection. All mice in this experiment were infected with a high inoculum of TcI-JR parasites (104). Data are expressed as mean ± SEM. (TIF) [file ppat.1009864.s004.tif]

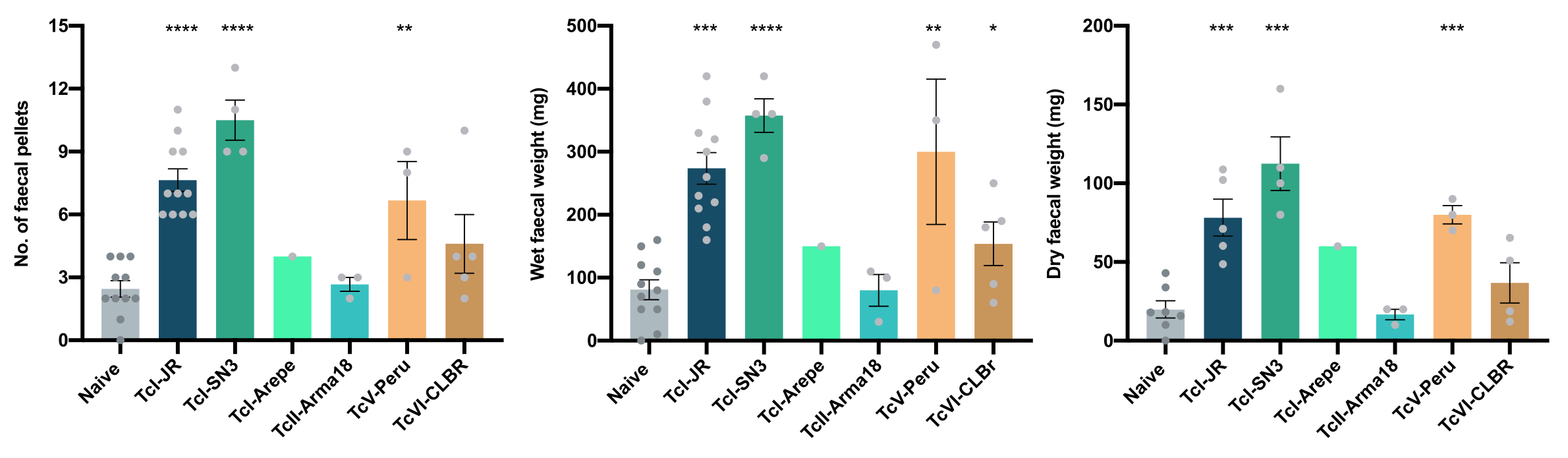

Supplement: S5 Fig — Faecal output analyses are expressed as faecal pellet count, wet and dry pellet weight at 30 weeks post-infection (p.i.) in the following groups: naive control (n = 7–11), TcI-JR (n = 5–11), TcI-SN3 (n = 4), TcI-ArePe (n = 1), TcIII-Arma18 (n = 3), TcVI-Peru (n = 3) and TcVI-CLBR (n = 4–5). (TIF) [file ppat.1009864.s005.tif]

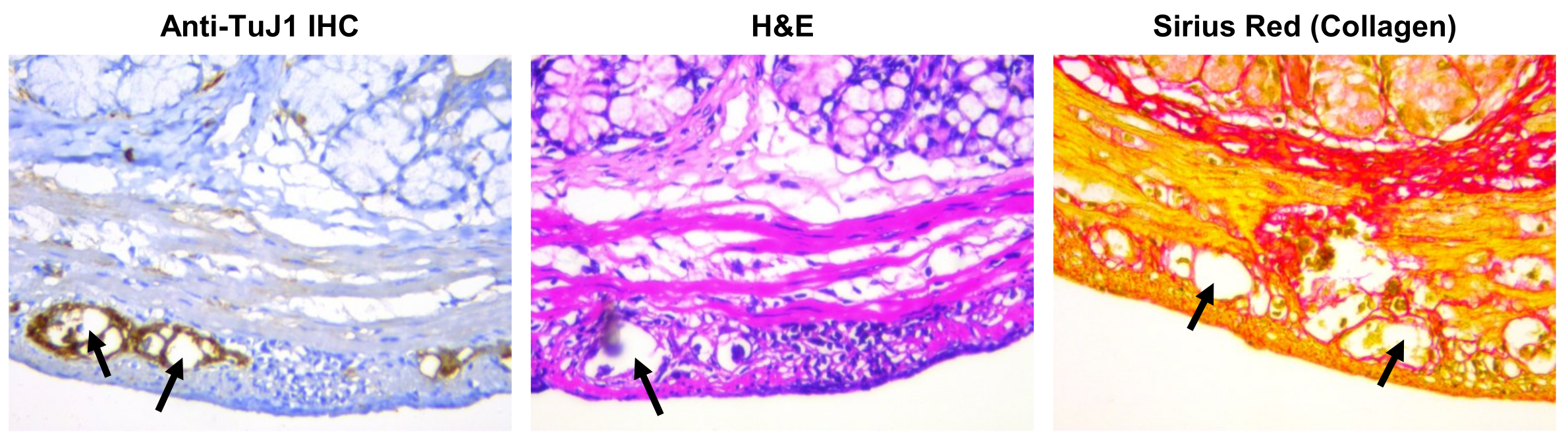

Supplement: S6 Fig — Colon tissue samples from C3H mice with chronic TcI-JR infections were cut as transverse cross-sections and images are oriented to show the mucosa above the smooth muscle layers and serosa at the lower edge. Acellular structures (arrows) within myenteric plexus ganglia that are refractory to staining by the indicated method. 400X magnification. (TIF) [file ppat.1009864.s006.tif]

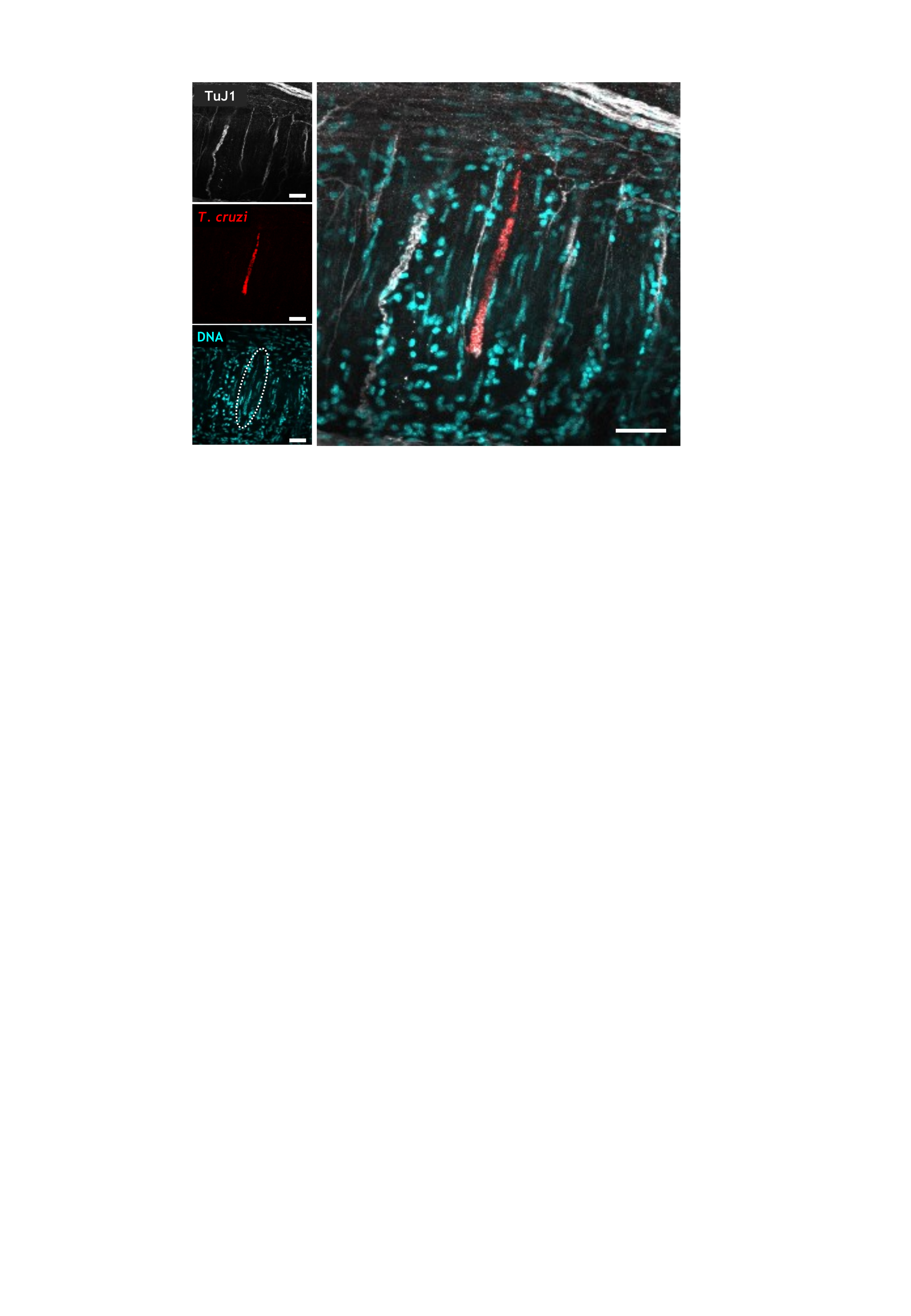

Supplement: S7 Fig — Representative immunofluorescent z-stack confocal images of whole-mount colonic muscularis from C3H mice chronically infected with fluorescent TcI-SN3 (mScarlet+) parasites. Image shows the localisation of TcI-SN3 parasites (red) in the submucosal layer of the ENS stained with anti-TuJ1 (white). DAPI (cyan) shows DNA and white circle indicates DNA of the parasite nest. Images were taken at 400X magnification, scale bar = 50 μm. (TIF) [file ppat.1009864.s007.tif]
