# Supplementary material for: Local association of Trypanosoma cruzi chronic infection foci and enteric neuropathic lesions at the tissue micro-domain scale
Source: PLoS Pathog. 2021 Aug 23;17(8):e1009864. doi: 10.1371/journal.ppat.1009864 (PMC8412264; doi:10.1371/journal.ppat.1009864)
Supplement: S1 Table — (DOCX) [file ppat.1009864.s008.docx]

| **Gene** | **Product** | **Forward Primer** | **Reverse Primer** | **Rationale** |
| --- | --- | --- | --- | --- |
| *Tubb3* | Neuron-specific tubulin β-3 (TuJ1) | CAGGGCCATTCTGGTGGACT | TAGTGCCCTTTGGCCCAGTT | Pan-neuronal marker |
| *Chat* | Choline acetyltranferase | GATGAACGCCTGCCTCCAAT | GGCATACCAGGCAGATGCAG | Encodes key enzyme involved in the biosynthesis of the excitatory neurotransmitter acetylcholine, released predominantly by the enteric excitatory muscle motor neuron and by other subsets of interneurons in the ENS. ChAt is also expressed by descending enteric neurons which are involved in peristalsis. |
| *SP* | Substance P | CCGACTGGTCCGACAGTGAC | CGCTTGCCCATTAATCCAAA | Encodes the excitatory neurotransmitter substance P, which is involved in the contraction of the enteric circular muscle during peristalsis. |
| *Nos1* | Neuronal nitric oxide synthase (nNOS) | ACTGACACCCTGCACCTGAAGA | GTGCGGACATCTTCTGACTTCC | *Nos1* encodes for the enzyme, nitric oxide synthase which catalyses the production of nitric oxide, the primary inhibitory neurotransmitter in enteric inhibitory neurons. *Nos2* and *Nos3* a encode for inducible and endothelial nitric oxide synthase. |
| *Nos2* | Inducible nitric oxide synthase (iNOS) | CAGCTGGGCTGTACAAACCTT | CATTGGAAGTGAAGCGTTTCG |  |
| *Nos3* | Endothelial nitric oxide synthase (eNOS) | CCTCGAGTAAAGAATTGGGAAGTG | AACTTCCTTGGAAACACCAGGG |  |
| *Ngfr* | Nerve growth factor receptor | GGTGATGGCAACCTCTACAGT | CCTCGTGGGTAAAGGAGTCTA | *Ngfr* encodes for the cell surface receptor of nerve growth factor; common receptor subunit for neurotrophins. |
| *NtrK1* | Neurotrophic tyrosine receptor kinase A | CGCTGAGTGCTACAACCTTC | GAAAGTCCTGCCGAGCATTC | Form receptor complexes with NGFR, which bind to neurotrophins and regulate proliferation, maintenance, survival and development of neurons in the ENS. |
| *NtrK2* | Neurotrophic tyrosine receptor kinase B | TTACGTGGGGCTGAGAAACC | TCCTGGACAAACTCGTCAGC |  |
| *NtrK3* | Neurotrophic tyrosine receptor kinase C | TACCTGGCTTCCCAGCACTTTG | GTGTCCTCCCACCCTGTAGTAATC |  |
| *Th* | Tyrosine hydroxylase | TCCTGCACTCCCTGTCAGAG | CACCGGCTGGTAGGTTTGAT | Encodes tyrosine hydroxylase, the rate-limiting enzyme in the biosynthesis of dopamine and other catecholamine neurotransmitters. |
| *Gapdh* | Glyceraldehyde 3-phosphate dehydrogenase | TCCTGCACCACCAACTGCTT | CACGCCACAGCTTTCCAGAG | Endogenous 'housekeeping' reference gene |
| *Vip* | Vasoactive intestinal peptide | AGGAGCAGGTGACCCTGACC | TCGCTGGTGAAAACTCCATCA | Encodes the neuropeptide VIP, one of the key inhibitory neurotransmitter involved in peristaltic reflex of the enteric smooth muscle. VIP is the primary neurotransmitter of noncholinergic secretomotor neuron and second for the enteric inhibitory muscle motor neuron which are involved in smooth mucsle peristaltic tone. |

**References**

- Akpan N, Caradonna K, Chuenkova MV, et al. (2008) Chagas' disease parasite-derived neurotrophic factor activates cholinergic gene expression in neuronal PC12 cells. *Brain Res* 1217: 195-202.
- Brookes SJ, Steele PA and Costa M. (1991) Identification and immunohistochemistry of cholinergic and non-cholinergic circular muscle motor neurons in the guinea-pig small intestine. *Neuroscience* 42: 863-878.
- Chevalier J, Derkinderen P, Gomes P, et al. (2008) Activity-dependent regulation of tyrosine hydroxylase expression in the enteric nervous system. *J Physiol* 586: 1963-1975.
- Chuenkova MV and PereiraPerrin M. (2004) Chagas' disease parasite promotes neuron survival and differentiation through TrkA nerve growth factor receptor. *J Neurochem* 91: 385-394.
- Costa M and Brookes SJ. (1994) The enteric nervous system. *Am J Gastroenterol* 89: S129-137.
- Costa M, Furness JB, Pompolo S, et al. (1992) Projections and chemical coding of neurons with immunoreactivity for nitric oxide synthase in the guinea-pig small intestine. *Neurosci Lett* 148: 121-125.
- Durand JL, Mukherjee S, Commodari F, et al. (2009) Role of NO synthase in the development of *Trypanosoma cruzi*-induced cardiomyopathy in mice. *Am J Trop Med Hyg* 80: 782-787.
- Esteban I, Levanti B, Garcia-Suarez O, et al. (1998) A neuronal subpopulation in the mammalian enteric nervous system expresses TrkA and TrkC neurotrophin receptor-like proteins. *Anat Rec* 251: 360-370.
- Furness JB. (2012) The enteric nervous system and neurogastroenterology. *Nature Reviews Gastroenterology & Hepatology* 9: 286-294.
- Furness JB and Costa M. (1982) Neurons with 5-hydroxytryptamine-like immunoreactivity in the enteric nervous system: their projections in the guinea-pig small intestine. *Neuroscience* 7: 341-349.
- Huang EJ and Reichardt LF. (2001) Neurotrophins: roles in neuronal development and function. *Annu Rev Neurosci* 24: 677-736.
- Li ZS, Pham TD, Tamir H, et al. (2004) Enteric dopaminergic neurons: definition, developmental lineage, and effects of extrinsic denervation. *J Neurosci* 24: 1330-1339.
- Maifrino LB, Liberti EA and de Souza RR. (1999) Vasoactive-intestinal-peptide- and substance-P-immunoreactive nerve fibres in the myenteric plexus of mouse colon during the chronic phase of *Trypanosoma cruzi* infection. *Ann Trop Med Parasitol* 93: 49-56.
- Weinkauf C and Pereiraperrin M. (2009) *Trypanosoma cruzi* promotes neuronal and glial cell survival through the neurotrophic receptor TrkC. *Infect Immun* 77: 1368-1375.
